# Comparative Analysis of the Serological Reactivity of Individuals with Clinical History of Malaria using Two Different ELISA Tests

**DOI:** 10.3390/diagnostics9040168

**Published:** 2019-10-30

**Authors:** Yorleydy Ruiz Moreno, Silvia Tavares Donato, Fátima Nogueira, Marcelo Sousa Silva

**Affiliations:** 1 Global Health and Tropical Medicine, Instituto de Higiene e Medicina Tropical, Universidade Nova de Lisboa, 1349-008 Lisbon, Portugal; yorleydy.moreno@ihmt.unl.pt (Y.R.M.); silviatdonato@gmail.com (S.T.D.); 2 Centro de Ciências Biológicas e da Saúde, Federal University of Campina Grande, 58439-900 Campina Grande, Brazil; 3 Global Health and Tropical Medicine, Instituto de Higiene e Medicina Tropical, Universidade Nova de Lisboa, 1349-008 Lisbon, Portugal; FNogueira@ihmt.unl.pt; 4 Immunoparasitology Laboratory, Department of Clinical and Toxicological Analysis, Federal University of Rio Grande do Norte, Natal 59012-570, Brazil

**Keywords:** *Plasmodium falciparum*, malaria, ELISA, serological diagnosis, serological markers

## Abstract

Early diagnosis of malaria reduces disease, prevents deaths, and contributes to decreased malaria transmission. The use of specific and sensitive antigens in the execution of serological diagnostics may have an impact on the transmission of the disease. However, many individuals cannot be easily diagnosed by serological tests due to low levels of antibodies in the serum. Using two different Enzyme-Linked Immunosorbent Assay (ELISA) tests (a commercial and an in-house ELISA), a total of 365 serum samples from individuals with a clinical history of malaria were analyzed. From the serum samples analyzed, 192 (53%) samples from the commercial ELISA and 219 (60%) samples from the in-house ELISA presented positive serological reactivity to malaria. The concordance of the samples tested (*n* = 365) between both ELISAs was of 67% (*n* = 242), and with the negative control was 100% (*n* = 17). We demonstrated that the in-house ELISA showed high antigenic reactivity to *Plasmodium falciparum* antigens when compared with the commercial ELISA. The degree of concordance of both ELISAs suggested the possibility of existence of other *P. falciparum* antigens present in the crude extract of *P. falciparum* that are important in the serological response during malaria infection.

## 1. Introduction

Early diagnosis and treatment of human malaria reduces disease, prevents deaths, and contributes to reduced transmission [1]. Detection of antibody response against malaria antigens is often done through Enzyme-Linked Immunosorbent Assay (ELISA) [2]; many of these diagnostic assays are designed with a single antigen or with recombinant antigens of different stages of the parasites (tissue infection or blood), where merozoite surface proteins can be detected, and have demonstrated good sensitivity and specificity [3]. However, many individuals cannot be easily diagnosed due to the low levels of antibodies present in their blood. In this context, the use of ELISA as a tool for laboratory diagnosis of malaria has some limitations, namely: (i) low antibody titres presented by individuals in the acute phase of infection; (ii) many *Plasmodium* sp. are involved in human malaria; (iii) different evolutionary forms of *Plasmodium* spp. during infection and life cycle; and (iv) different resistance and susceptibility profiles during infection, and consequently in the immunity profile against malaria parasites.

Associations among antibodies against parasite antigens and malaria risk areas are not always consistent [4]; some may depend on parasite antigens and other considerations may be due to the endemic areas of malaria [5]. It should be considered that single responses of antibodies against antigens might be inadequate to for use as biomarkers and indicators of malaria transmission intensity [6]. Other authors have shown that new serological biomarkers can more accurately estimate the recent exposure to *P. falciparum* not only of a community, but also for several communities [7,8]. In 2015, approximately 3.2 billion people—nearly half of the world’s population—were at risk of malaria, mainly by *P. falciparum* [9,10].

After several decades of disease control campaigns, malaria persists as one of the most serious public health problems, not only in endemic countries, but also in non-endemic regions, where the number of cases of imported malaria via tourists and immigrants is on the rise [11]. *P. falciparum* is one the most lethal species of the malaria parasites that infect humans [12], and is responsible for the highest number of serious pathologies associated with the disease [13].

In this work, we performed a comparative analysis of the serological reactivity of total antimalarial antibodies obtained by a commercial ELISA using recombinant antigens from *Plasmodium* sp., compared to an in-house ELISA, using crude extract of *P. falciparum* 3D7. Thus, we showed that several other *P. falciparum* antigens are equally important for the serological diagnosis of malaria, as well as some recombinant antigens commonly used in commercial ELISA.

## 2. Materials and Methods

### 2.1. Study Design

In this work, a concordance analysis was performed between two ELISA tests, aiming to demonstrate that crude extract of *P. falciparum* contains proteins with antigenic activity capable of reacting with antibodies produced during acute human malaria. To perform this analysis, 365 serum samples from individuals with a clinical history of malaria were analyzed by an in-house ELISA and also by a commercial ELISA test (ELISA EIA Kit, Bio-Rad, Marnes-la-Coquette, France) used for serological diagnosis of human malaria. Serum samples from individuals without clinical history of malaria (*n* = 17) were used as a negative control. The results were categorized as reactive and non-reactive [14]. Serum samples were obtained from the Clinical Unit for Tropical Diseases of the Institute of Hygiene and Tropical Medicine (IHMT) from New University of Lisbon (UNL). Samples were obtained from individuals diagnosed with a clinical history of malaria and subsequently submitted to serological diagnosis of malaria at the IHMT-UNL. Authorization for the use of serum samples from the individuals submitted to serological diagnosis of malaria was obtained and approved by the standards of the IHMT-UNL institutional ethics committee (approval ref 4-2012-PN of CE-IHMT-UNL, February 22, 2012). All serum samples were used exclusively for laboratory diagnosis of malaria by immunoassays.

### 2.2. Plasmodium falciparum Parasite, Cell Culture, and Crude Protein Extract

To perform serological analyses, *P. falciparum* 3D7 strain blood-stage parasites were used to produce soluble crude antigen extract (*P. falciparum* extract). *P. falciparum* parasites were cultured using modifications to the method described by Trager and Jensen [15]. Briefly, parasites were grown in human erythrocytes in RPMI 1640 medium containing L-glutamine (ThermoFisher Scientific, Carlsbad, CA) supplemented with 5% (*v*/*v*) human serum and 5% (*v*/*v*) Albumax II (ThermoFisher Scientific, Carlsbad, CA), and incubated at 35 °C in a humid atmosphere with 5% CO_2_.

Total crude protein of *P. falciparum* was determined according to the method described by Medina et al., 2013 [16], with some adjustments. When erythrocytes evidenced parasitemia above 10% and showed a predominance of merozoites and schizonts, cultures were transferred to 15 mL tubes, homogenized, and centrifuged (centrifuge 5810r, Eppendorf, Damstadt, Germany) at 2000× *g* for 5 min at room temperature (RT). Supernatant was discarded, and the pellet was resuspended in a volume of saponine 0.05% that corresponded to 5 times the pellet volume. Tubes were placed on ice for 15 min and then centrifuged at 3220× *g* at 4 °C for 20 min. Supernatant was discarded and the pellet was washed three times with cold phosphate buffered saline (PBS) and centrifuged at 18,000× *g* for 10 min at 4 °C. For the lysis of the parasites, 4 times the pellet volume of lysis buffer with inhibitor was added (0.1% Triton X-100 in PBS with protease inhibitor (cOmplete ULTRA Tablets, Roche, Switzerland)). The tubes were placed on ice for 30 min under agitation, homogenizing every 10 min in the vortex. Pellets containing parasites were frozen at −70 °C for 5 min and then heated at 37 °C for 5 min. After three freeze–thaw cycles, samples were then centrifuged at 18,000× *g* for 10 min at 4 °C. The supernatant was recovered, and the pellets containing hemozoin were discarded. Soluble protein extract of *P. falciparum* was cryopreserved at −20 °C.

The MicroBCA method was used for protein quantification of *P. falciparum* extract. The entire procedure was performed according to the manufacturer’s instructions (MicroBCA™ Protein Assay Reagent Kit, ThermoFisher Scientific, Rockford, USA), using Brand plates^®^-pureGrade ™ (Brand, Germany) [17]. For the calibration curve, 225 μL of the prepared solution was added into the wells of the plate (BRANDplates microplates, Brand, Germany) according to the desired concentration. For the samples to be quantified, 200 μL of the working reagent (supplied by the manufacturer) and 25 μL of sample were added and homogenized. The plate was incubated for 30 min at 37 °C. The results were read on a microplate reader (microplate reader model 680, BioRad) at 562 nm.

### 2.3. Reactivity of Recombinant Proteins of Plasmodium sp. to Total Antibodies in Sera from Individuals with Clinical History of Malaria using Commercial ELISA

A commercial ELISA was performed to detect total anti-*Plasmodium* spp. antibodies (Malaria EIA kit, BioRad). This commercial assay is an indirect ELISA that detects, in human serum or plasma, specific anti-*P. falciparum, -P. vivax, -P. ovalae*, and -*P. malariae* IgM, IgG, and IgA antibodies at any stage of the parasitic life cycle. This assay uses recombinant antigens of *P. vivax*, *P. falciparum, P. ovalae*, and *P. malariae*. All sample processing was carried out according to the manufacturer’s instructions.

Briefly, 50 μL of undiluted of serum samples was deposited in the wells of a 96 well plate (Microtiter 96 well plates, Thermo Fisher Scientific, Rochester, NY, USA) The plate was incubated for 30 min at 37 °C in an orbital shaker (Bio-Rad Microtiter/Plate Shaker). After incubation, the plate was washed five times with 50 μL/well of working strength wash and 50 µL of the secondary antibody was added (secondary antibody conjugated to horseradish peroxidase with buffered saline containing surfactant and stabilizers) to each well. Since the substrate is photosensitive, the plate was incubated at room temperature for 30 min in the dark. The plate was washed five times with working strength wash, 50 µL substrate was added (urea peroxide and tetramethylbenzidine), and the plate was incubated at room temperature for 30 min. The plate was protected from light during this incubation. Finally, 50 μL of stop solution (0.5 M H_2_SO_4_) was added to each well (blue color changed to yellow), and OD was measured at 450 nm on a microplate reader (Bio-Rad Model 680 Microplate Reader). Antibody quantification in serum samples was calculated by cut-off value. The cut-off value supplied by the manufacturer was determined by the following formula:

$$\frac{NC1+NC2+NC3}{3}+0.1$$

where NC is the optical density (OD) value of negative controls and 0.1 is a factor determined by the manufacturer. Thus, samples with ODs lower than the cut-off value were considered negative and samples with ODs values greater than thecut-off value were considered positive.

### 2.4. Reactivity of Plasmodium falciparum Crude Extract to Total Antibodies in Sera from Individuals with Clinical History of Malaria using In-House ELISA

In this study, ELISA was used to analyze the ability of *P. falciparum* antigens to be recognized by total antibodies (IgM, IgG, and IgA) in sera from individuals with clinical history of malaria. First, 96 well microtiter plates (Thermo Fisher Scientific, Rochester, NY, USA) were coated with different concentrations (from 10 to 100 ng/well) of *P. falciparum* 3D7 crude extract diluted in 0.1 M bicarbonate buffer, pH 8.5, and incubated overnight at 4 °C. Plates were washed three times with 200 μL/well of wash buffer (PBS with 0.05% Tween-20), blocked with 200 μL/well of blocking buffer (PBS with 5% of skimmed milk), and incubated at room temperature for 1 h. Plates were then washed thrice. The serum samples (1:200 dilution) were diluted in antibody buffer (washing buffer with 1% of skimmed milk) and added to the plates (100 μL/well). Plates were incubated for 1 h at room temperature in an orbital shaker to avoid non-specific binding. Afterward, plates were again washed three times and the secondary anti-human antibody conjugated to alkaline phosphatase (Calbiochem) was added. Conjugated antibody was diluted 1:10,000 (*v*/*v*) in antibody buffer according to manufacturer’s recommendations, and 100 μL/well added to plate. After 1 h, the plates were washed five times for 10 min and incubated with 100 μL of substrate solution (20 mg of 4-nitrophenyl phosphate disodium salt hexahydrate diluted in 20 mL of water) for 30 min at room temperature, protected from light. Finally, 50 μL/well of stop solution (3 N sodium hydroxide) was added to stop the reaction. The absorbance (OD) was obtained at 405 nm in a microplate reader (Bio-Rad Model 680 Microplate Reader).

### 2.5. Statistical Analysis

Statistics analysis was carried out using Prism 6.0 for Windows (GraphPad Software, Inc., San Diego, USA) and MedCalc. Ink (Statistic Software MedCalc, Ostend, Belgium). Kruskal–Wallis and chi-square tests (χ^2^) were used to evaluate the associations among continuous and categorical variables. Differences in medians of the study population data were calculated using the parametric student test, or normalized transformations were performed on raw data before testing by one-way analysis of variance (ANOVA). Receiver operating characteristic (ROC) curves were generated using GraphPad Prism (version 6.00 for Windows, GraphPad Software, San Diego California USA). A 95% confidence interval was considered for the area under the ROC curve.

Cut-off values were inferred from these curves for each ELISA by choosing the best compromise between sensitivity and specificity. Positive predictive value, negative predictive value, and Youden index were calculated. The best positivity point between ELISA assays, sensibility, and specificity to each ELISA was calculated as a function of cut-off value. Correlation between the two ELISAs was tested by the Bland–Altman test and Pearson product–moment correlation coefficient, and *p* values < 0.05 indicate a statistically significant correlation. The data were analyzed to 95% statistical significance (*p* < 0.05).

## 3. Results

**Figure 1:**
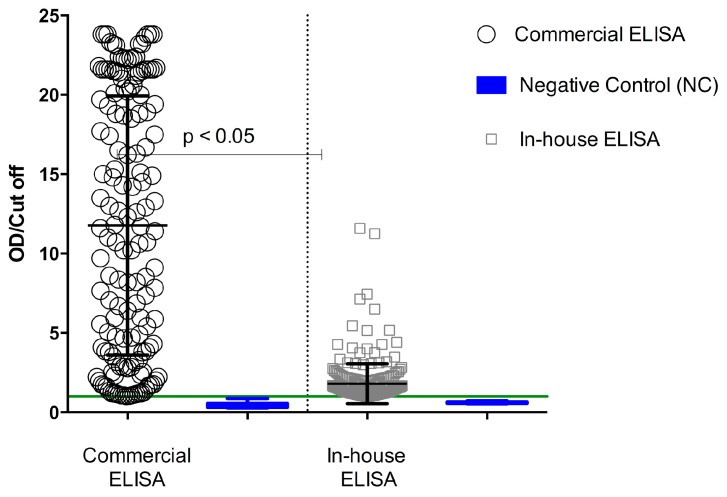
Serological reactivity of in-house and commercial Enzyme-Linked Immunosorbent Assays (ELISAs). Serum samples were evaluated by commercial ELISA incorporating recombinant antigens of *P. vivax*, *P. falciparum*, *P. malariae*, and *P. ovalae*; and by in-house ELISA (*P. falciparum* crude extract). Sera of individuals exposed to *Plasmodium* sp. (*n* = 365) and not exposed to the parasite (negative control, NC *n* = 17) were evaluated in parallel by the two ELISAs. The results were analyzed by Student’s *t*-test and have been represented by scatter dot plot, medians, and interquartile ranges. Green line represents cut-off.

**Figure 2:**
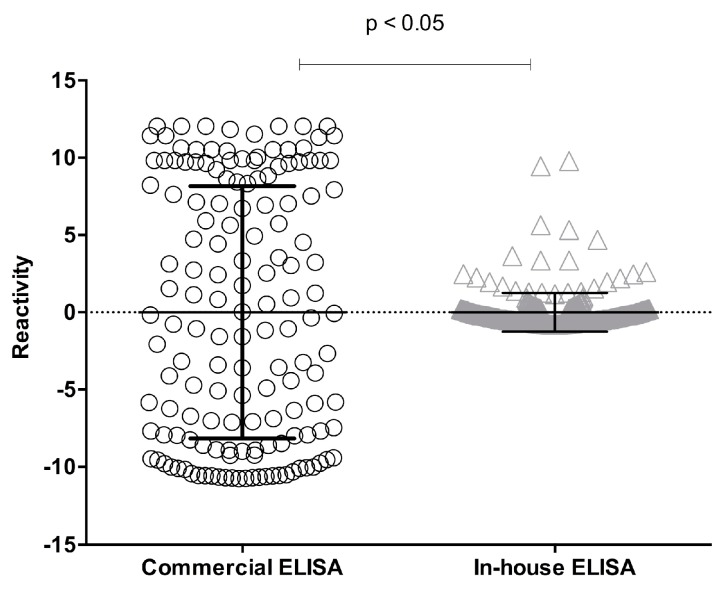
Comparison of serological reactivity between the two ELISAs. Serological reactivity of crude extract of *P. falciparum* to total anti-*P. falciparum* antibodies was evaluated by commercial and in-house ELISAs using sera from individuals exposed to *Plasmodium* sp. (*n* = 365). Results were analyzed by Student’s *t*-test and have represented by whisker boxes, medians, and interquartile ranges, with a representation of the statistical value (*p* < 0.05).

The capacity of *P. falciparum* 3D7 crude extract to recognize antibodies anti-*P. falciparum* by an in-house ELISA was compared with results obtained from a commercial ELISA, which incorporates recombinant antigens of *P. vivax*, *P. falciparum*, *P. malariae*, and *P. ovalae*. The degree of concordance of the two ELISAs, suggesting the possibility of other important antigens being involved in the serological response during human malaria, indicated that crude antigens were more reactive to ELISA than recombinant proteins. Using the commercial ELISA, increased antibody reactivity was observed in the serum samples, reaching maximum values of about 24 OD/cut-off, whereas using in-house ELISA, the same sample achieved OD/cut-off maximum values around 14 (Figure 1). Serological reactivity of total antibodies against *P. falciparum* was of 53% for the commercial ELISA and 60% for the in-house ELISA (Table 1). A paired analysis of samples fulfilling the acceptance criteria for both methods showed a significant difference (*p* < 0.05; Student’s *t*-test) (Figure 2).

Sera from individuals not exposed to the parasite (control group *n* = 17) were identified as non-reactive by in-house and commercial ELISA, indicating a 100% of concordance. However, sera from individuals exposed to *Plasmodium* sp. (*n* = 365) presented 67% (*n* = 242) concordance. The agreement between both ELISAs for the sera of individuals exposed to *Plasmodium* sp. was 40% (*n =* 144) of positive sera and 27% (*n* = 98) of negative sera samples, representing a disagreement of 33% (Table 1).

**Figure 3:**
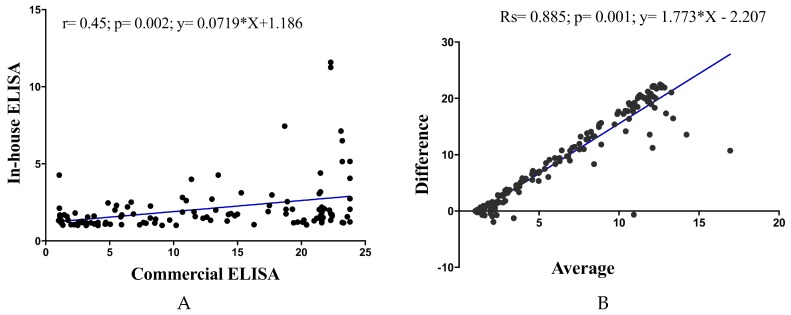
Correlation analysis between commercial and in-house ELISAs. Pearson correlation plots evaluated correlation between commercial and in-house ELISAs (**A**) and Bland–Altman plots (**B**). Blue line represents linear regression of data. Pearson correlation analysis showed a significant positive correlation of in-house versus commercial ELISAs (Pearson *r* = 0.45; *p* = 0.002; *y* = 0.07179 × X + 1.186). Bland–Altman plots showed significant correlation between the two ELISAs (Rs = 0.885; *p* = 0.001; *y* =1.773 × X − 2.207). *p* value < 0.05 indicates a statistically significant correlation.

Pearson correlation analysis showed a significant positive correlation of in-house ELISA versus commercial ELISA (Pearson *r* = 0.45; *p* = 0.02; *y* = 0.07179 × X + 1.186). Examination by Bland–Altman analysis showed a good agreement across the range of samples tested, with a bias of less than 5% between the ELISA readings (*R* square; Rs = 0.885; *p* = 0.01; *y* = 1.773 × X − 2.207) (Figure 3).

**Figure 4:**
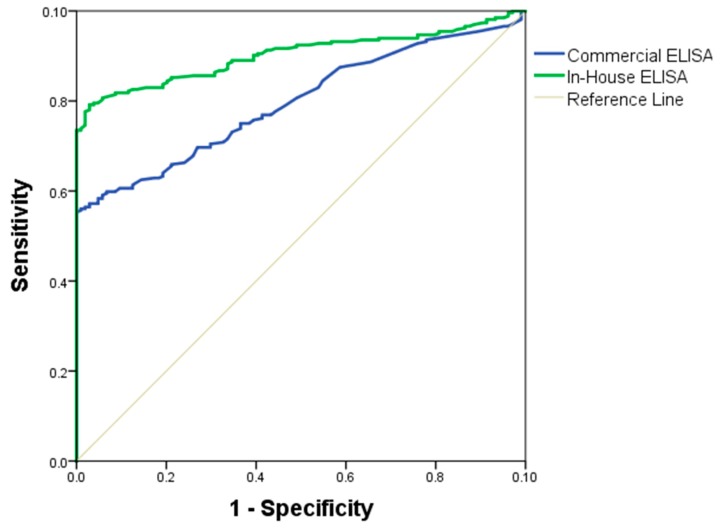
ELISA receiver operating characteristic (ROC) curves. Absorbance of sera samples obtained by in-house and commercial ELISAs were plotted and ROC curves generated. Blue line represents the sensitivity to commercial ELISA, green line represents the sensitivity of in-house ELISA, and black line represents the specificity. The area under the curve was determined with a 95% confidence interval.

The quality of the in-house and commercial ELISAs was expressed through a ROC curve. The area under the ROC curve measures the accuracy. An area of 1 represents a perfect test; an area of 0.5 represents a worthless test. A rough guide to classify the accuracy of a diagnostic test is the traditional academic point system: 90–100 = excellent, 80–90 = good, 70–80 = fair, 60–70 = poor, 50–60 = fail. The area under the ROC curve for the in-house ELISA was of 89% (95% IC = 0.8464–0.9267, SD = 0.0204) and for the commercial ELISA was 71% (95% IC = 0.663–0.757, SD = 0.0267) (Figure 4). Antigen performance was evaluated by the following parameters: area under the curve (Table 2), diagnosis parameters showing a sensitivity (75%), specificity (57%), positive predictive value (VPP = 66%), and negative predictive value (NPV = 68%), presented in the Table 3. The formulas used to obtain these variables were:

Sensitivity: PV/ (PV + FN)
Specificity: NV/ (NV + FP)
Positive Predictive Value: PV/ (PV + FP)
Negative Predictive Value: NV/ (NV + FN)

where PV is the positive value, NV is the negative value, FN is false negative value, and FP is false positive value.

## 4. Discussion

In this study, an in-house ELISA was evaluated. A crude extract of *P. falciparum* 3D7 was used as antigen. This antigen was constituted of proteins extracted from merozoite, gametocyte, and trophozoite forms; this characteristic could possibly contribute to a better recognition of the antimalarial antibodies. A similar study was carried out in Senegal to understand the humoral immune response in *P. falciparum* infection and in their ELISA. They also used mainly merozoite antigens (MSP1p19, Pf13-DBL1a) and obtained good seroprevalence responses [3,18].

A concordance analysis between the two ELISAs was made. This analysis was done to detect whether proteins or protein fractions present in the antigen had the capacity to recognize anti-*P. falciparum* antibodies, and to see whether *P. falciparum* crude extract was more or less reactive than recombinant proteins of commercial ELISA. The results suggested that the commercial ELISA, which was constituted of recombinant proteins, had a higher cut-off but identified a lower number of positive serum samples. On the other hand, the in-house ELISA had a lower cut-off, but identified a greater number of positive serum samples. These findings suggest that crude extract of *P. falciparum* contains a diverse protein from different parasite forms capable of recognizing a greater number of antimalarial antibodies. In another study, a comparative analysis using two types of antigens (PfAMA-1 and PfMSP1) alone and combined was performed, concluding that the use of several antigens in diagnostic testing presented greater serological reactivity (84-87%) than when a single antigen is used [19,20].

This study indicated that in-house ELISA and commercial ELISA are suitable to detect anti-*P. falciparum* antibodies, since they showed a high level of correlation and concordance, supporting the clinical use of either. However, the variability observed among the ELISA protocols and among the number of reactive samples suggests that the antigen response in the in-house ELISA was more complex, probably because it contained a greater number of immunogenic proteins capable of recognizing a higher number of antibodies against *P. falciparum*. In addition, the concentration of *P. falciparum* antigens used for ELISA plate adsorption is an important step in the process of optimizing an immunoassay for the determination of antimalarial antibodies. In this study, serological reactivity for antimalarial antibodies was evaluated by ELISA using different concentrations of the total protein of *P. falciparum* (from 10 to 100 ng/well). Here, the *P. falciparum* extract concentration considered appropriate for the ELISA was 100 ng/well, since the best serological reactivity (absorbance) was obtained at this concentration (results presented in supplementary data). It is also known that excess antigen adsorbed to the ELISA plate may cause an “antigen stacking” phenomenon, resulting in the elimination of antigen–antibody complex during the wash steps of the assay. Therefore, a total *P. falciparum* antigens concentration of 100 ng/well was considered in this study. In-house ELISA is also a technique that could minimize cost difficulties, since it uses reagents prepared in house and it is an easy technique to execute, making it closer to a rapid test or point-of-care test (POCT), and therefore a useful diagnostic tool for malaria.

When trying to evaluate complex antigens, it is also important to evaluate the assay conditions (incubation and temperature) and to use antibodies in sera from different geographic areas in order to define the best conditions under which results with good sensitivity might be obtained. In this study, a dilution of conjugated antibodies was used according to the manufacturer’s standards. Serum samples were diluted according to the availability of biological material, optimizing a 1/200 (*v*/*v*) dilution of all samples tested. In addition, increasing the incubation temperature from 5 to 37 °C decreased the affinity of the antigen–antibody complexes by decreasing the stability of the coupling complex [21,22].

In our in-house ELISA, antigens were incubated at 4 °C overnight, unlike the commercial ELISA antigen, which was incubated at 37 °C for 2 h. Studies carried out by Lipschultz et al. (2002) and Reverberi et al. (2007) concluded that the incubation temperature influences the antigen–antibody affinity, because it decreases the affinity of antigen–antibody complexes by declining the stability of the coupling complex [21,23]. Therefore, temperature variations could influence the antigenic reactivity and affect the performance of serological assays. In another study, it was shown that in ELISA samples, incubation at 4 °C overnight gave better performance for the measurement of antigen reactivity, because the antibody was strongly bound to the antigen, and there was evidence of increased serological reactivity [24]. Moreno et al. (2011) found that soluble antigen rhoptry neck protein 1 (PvRON1) of *P. vivax* incubated at 4 °C presented better antigenicity when it was incubated at 37 °C [25]. Previous studies have concluded that the temperature of incubation influences the reversible antigen–antibody kinetics by changing the constant association/dissociation equilibrium [23], which may affect the sensitivity of the assay [26]. The use of specific and sensitive antigens in the execution of diagnostic serological tests may have an impact on malaria transmission. Other authors have been interested in evaluating the specific response of antigens–antibodies to *Plasmodium* sp. exposure involved in the innate and humoral immune response [20]. It is important to remember that many antigens present in the pre-erythrocyte and erythrocyte stages play important roles in the protection against malaria [3,27,28]. Several studies have been carried out on the functionality of *P. falciparum* antigens in diagnosis and it has been found, for example, that MRCAg1 chimeric antigen, containing eight epitopes of *P. falciparum*, is a potential marker for the detection of malaria caused by *P. falciparum* [29,30,31]. In addition, some studies have been conducted to understand the antibody response against malaria infection using ELISA [2,3], suggesting that ELISA is a good method for evaluating the humoral immune response. However, for the rapid and efficient diagnosis of human malaria by immunoassays, it is necessary to develop faster, more specific, and more sensitive tests.

## 5. Conclusions

The results suggest that in-house ELISA is a good technique for the detection of anti-*P. falciparum* antibodies. *P. falciparum* crude extract includes proteins that recognize the antibodies present in sera of individuals with clinical history of malaria. The results also suggest that the crude extract of *P. falciparum* contained protein or protein fractions with immunogenic activity, which could be used as diagnostic in context of human malaria. This analysis is important because endemic malaria communities would benefit from having more extensive information on the antigenic reactivity of *P. falciparum* that could be used in the developing of new immunoassays.

## Figures and Tables

**Table 1:** Comparative analysis and concordance between commercial and in-house ELISAs.

Results	Reactive		Non-Reactive		Negative Control	
	*n*	%	*n*	%	*n*	%
Commercial ELISA	192	53	173	47	17	100
In-House ELISA	219	60	146	40	17	100
Both ELISAs	144	40	98	27		

**Table 2:** Parameters of area under curve (AUC)—ROC curves of commercial ELISA and in-house ELISAs.

Test	AUC	S	95% CI	*p*-Value
**ELISA commercial**	0.712	0.0267	0.663 to 0.757	<0.0001
**ELISA in-house**	0.891	0.0203	0.876 to 0.937	<0.0001

AUC: area under the curve; confidence interval (CI) = 95%; *p*-value: significance level; S: sensitivity.

**Table 3:** Serological reactivity parameters used for the determination of anti-*Plasmodium falciparum* antibodies by in-house ELISA.

Parameters	Value	CI 95%
Sensitivity	75%	(0.6826–0.8095)
Specificity	57%	(0.4891–0.6415)
Positive Predictive Value (PPV)	66%	(0.5906–0.7201)
Negative Predictive Value (NPV)	68%	(0.5887–0.7467)
Youden index J	0.873	
AUC	0.886	

AUC (Area Under the Curve); CI = 95% (Confidence Interval).

## Supplementary Materials

Supplementary File 1
