# Supplementary material for: Comparative Analysis of the Serological Reactivity of Individuals with Clinical History of Malaria using Two Different ELISA Tests
Source: Diagnostics (Basel). 2019 Oct 30;9(4):168. doi: 10.3390/diagnostics9040168 (PMC6963549; doi:10.3390/diagnostics9040168)
Supplement: Supplementary File 1 [file diagnostics-09-00168-s001.pdf]

Supplementary data: Total IgG anti-*Plasmodium falciparum* antibodies in serum samples from individuals with malaria. Different concentrations (from 10 to 100 ng/ well) of a total *Plasmodium falciparum* protein extract were adsorbed to a 96-well ELISA microplate.

Plasmodium falciparum **Protein Extraction (ng protein/ well)**	**Samples ID and Absorbance (450 nm)**											
	**PC17**	**PC18**	**PC19**	**PC20**	**S194**	**S195**	**S196**	**S198**	**S199**	**S200**	**S201**	**S202**
**100**	**1,057**	**0,921**	**0,839**	**0,831**	**1 027**	**0.977**	**1 593**	**0.871**	**1 132**	**0.803**	**0.887**	**1 008**
50	0,892	0,771	0,696	0,733	0.788	0.772	1 167	0.773	0.802	0.727	0.785	0.831
20	0,846	0,778	0,611	0,586	0.785	0.881	0.992	0.751	0.767	0.712	0.736	0.788
10	0.704	0,751	0,614	0,545	0.667	0.727	0.830	0.634	0.704	0.626	0.691	0.735

PC: Positive control - serum samples obtained from commercial ELISA kit (ELISA EIA Kit, Bio-Rad, USA); **S:** Serum samples obtained from individuals with clinical history of acute malaria; **Serum samples dilution:** 1/200 (v/v).
